# Case Report: Adenylosuccinate lyase deficiency type I caused by splicing disruption due to a novel missense variant in the *ADSL* gene

**DOI:** 10.3389/fgene.2025.1670299

**Published:** 2025-10-24

**Authors:** Artem Borovikov, Ksenia Davydenko, Aysylu Murtazina, Artem Sharkov, Ilya Kanivets, Alexandra Filatova, Mikhail Skoblov

**Affiliations:** ^1^ Research Centre for Medical Genetics, Moscow, Russia; ^2^ Veltischev Research and Clinical Institute for Pediatrics and Pediatric Surgery of the Pirogov Russian National Research Medical University, Moscow, Russia; ^3^ Genomed, Moscow, Russia; ^4^ FSBEI FPE RMACPE MOH, Moscow, Russia

**Keywords:** ADSL, ALD, adenylosuccinate lyase deficiency, case report, splicing

## Abstract

Adenylosuccinate lyase deficiency (ALD) is a rare neurometabolic disorder caused by biallelic loss-of-function variants in the ADSL gene. We report a severe type I ALD case involving a 2-year-old boy presenting with early-onset polymorphic seizures (clonic/myoclonic), developmental delay, and progressive neurological deterioration. Seizures were temporarily controlled with ethosuximide and vigabatrin, though neurodegeneration progressed. Analysis of whole-exome sequencing data revealed compound-heterozygous variants in the ADSL gene: the known pathogenic missense variant c.340T>C (p.Tyr114His) and a novel variant c.859A>G (p.Ile287Val). Although p.Ile287Val is predicted to be benign at the protein level, RNA analysis demonstrated that c.859A>G activates a cryptic splice site in exon 8, resulting in aberrant transcripts (64%, 4-bp deletion, targeted by nonsense-mediated decay) and a smaller proportion of normal transcripts (36%) encoding the p.Ile287Val protein. This case highlights splicing disruption as a novel pathogenic mechanism in ALD and expands the mutational spectrum associated with the disease. This case also underscores the importance of integrating RNA analysis with genomic data to uncover cryptic splicing defects, especially when protein-level predictions suggest benignity.

## 1 Introduction

Adenylosuccinate lyase deficiency (ALD; OMIM 103050) is a rare autosomal recessive disorder caused by the accumulation of neurotoxic metabolites (S-Ado and SAICAr), impairing early development (Spiegel et al., 2006; Dutto et al., 2022). ALD is commonly classified into type I (severe, early-onset form), type II (moderate form with developmental delay and seizures), and a rare, fatal neonatal form, with severity generally correlating with residual enzymatic activity (Kmoch et al., 2000; Jurecka et al., 2008). While the majority of previously reported ADSL variants are missense mutations studied primarily for their protein-level effects (Kmoch et al., 2000; Jurecka et al., 2008; Zikanova et al., 2010), we identified the first disease-associated variant, c.859A>G (p.Ile287Val) [Clinvar ID: 1437877], that exerts pathogenicity through splicing disruption and nonsense-mediated decay (NMD), revealing an alternative disease mechanism in ALD.

## 2 Materials and methods

### 2.1 Molecular genetic diagnostics

Blood samples were collected from the proband and his unaffected parents. Genomic DNA was extracted using standard methods. DNA diagnosis in the proband was carried out by targeted high-throughput sequencing (HTS) of clinically relevant genes (clinical exome sequencing, CES). CES was performed on an Illumina NextSeq 500 instrument in the 2 × 151 bp paired-end mode. A total of 13.7 million reads were obtained, corresponding to a 99.9× on-target average sequencing depth, based on the TruSight One Sequencing Panel target region list. The raw sequencing data have been processed with a previously published custom pipeline (Marakhonov et al., 2018). The mapped reads were visualized using the Integrative Genomics Viewer (IGV) software (https://www.igv.org/, accessed on 20 November 2023; ^©^ 2013–2018 Broad Institute, Boston, MA, United States, and the Regents of the University of California, San Diego, CA, United States). Variant filtering was based on their frequency, with variants having a frequency of less than 1% in the Genome Aggregation Database (gnomAD v.2.1.1) and coding region sequence effects such as missense, nonsense, coding indels, and splice sites being considered. The clinical significance of the variants was evaluated using the ACMG criteria for variant interpretation. All variants are reported according to the NM_000026.4 transcript of the *ADSL* gene.

### 2.2 RT-PCR analysis

To obtain mRNA, peripheral blood mononuclear cells (PBMCs) were separated from the whole blood by a density gradient centrifugation method using Ficoll. Then, cells were lysed in ExtractRNA buffer (Eurogen, Russia), аnd total RNA was prepared by phenol-chloroform extraction. For purification from genomic DNA, the RNA was treated with DNase I (Thermo Fisher Scientific, Waltham, MA, United States). cDNA was obtained using a reverse transcription system (Dialat Ltd., Moscow, Russia). To assess the quality of the cDNA, qPCR was performed with primers for the B2M housekeeping gene. PCR amplification was performed with the following primers: ADSL Ex5F (5′-ATC​TCC​AGA​ACT​TGA​AGC​GTG) and ADSL Ex9R (5′-TGG​GAT​TCC​GCT​TAT​ATG​GCA). The PCR product was analyzed on a 2% agarose gel with subsequent Sanger sequencing. To assess the ratio of mRNA isoforms, fragment analysis with a FAM-6-tagged forward primer was performed.

### 2.3 NMD inhibition

In order to inhibit the nonsense-mediated mRNA decay, patient PBMCs were obtained under sterile conditions and treated with 300 μM cycloheximide. After a 6-h incubation, mRNA was isolated and processed as described above.

### 2.4 *In vitro* splicing analysis

*In vitro* splicing analysis was performed using the minigene expression system as described by Davydenko et al. (2024). For minigene construction, the region encompassing exons 8 and 9 of the ADSL was amplified from the genomic DNA of the heterozygous carrier with the following primers: EcoRI-ADSL Ex8F (5′-AAA​AGA​ATT​CTT​CTT​TCC​ATG​ATG​CCT​TAA​GC) and XhoI-ADSL Ex9R (5′-AAA​ACT​CGA​GAA​CTC​CCT​CAA​GTG​TGT​TTT​CT). The obtained wild-type and mutant PCR products were cloned into the pSPL3-Flu2 plasmid vector. The structure of minigenes was confirmed by sequencing.

The vectors were then transfected into HEK293T cells by calcium phosphate transfection. The cells were harvested after 48 h, and total RNA and cDNA were obtained in the same way for the mRNA analysis. To detect the splicing products, a plasmid-specific primer, TurboFP-F (5′-ACA​AAG​AGA​CCT​ACG​TCG​AGC​A), and an *ADSL* gene-specific primer, Ex9R (5′-TGG​GAT​TCC​GCT​TAT​ATG​GCA), were used. The PCR product was analyzed in a urea polyacrylamide gel with urea and further Sanger sequencing. The ratio of mRNA isoforms was calculated by fragment analysis.

### 2.5 3D Modeling of protein structure

Structural assessment and visualization of the ADSL protein, including wild-type and mutant forms, were performed using the SWISS-MODEL structure assessment web server (https://swissmodel.expasy.org/assess) (Waterhouse et al., 2024). Mol* viewer (https://molstar.org/viewer) was used for 3D visualization and alignment of wild-type and mutant proteins (Sehnal et al., 2021).

## 3 Results

### 3.1 Clinical presentation

The proband was a 2-year-old boy (the first known affected family member) with a history of clonic, myoclonic, and generalized seizures. He was born at term following his mother’s first pregnancy, with a birth weight of 3,500 g, a body length of 52 cm, and Apgar scores of 8 and 9 at 1 min and 5 min, respectively. By the end of the first month, he exhibited poor sucking reflexes and minimal weight gain (only 90 g). Seizures began at 1 month of age, characterized by clonic, myoclonic, and generalized seizures, occurring up to five times daily. During the first 2 years of life, the patient experienced asymmetric tonic seizures, spasms, and eyelid myoclonias. At 2 years of age, the proband’s developmental milestones remained below normal (height: 79 cm [−2.35 SD], weight: 9.5 kg [−2.60 SD], head circumference: 45 cm [−2.56 SD]). A clinical examination showed muscular dystonia, hyperreflexia in both upper and lower limbs, and hypersalivation.

Video electroencephalography (EEG) at 1 month of age showed the absence of a posterior dominant rhythm, but alpha and theta waves were dominant in the background. Physiological sleep patterns were formed and stable. Low-index, multiregional epileptiform activity was recorded in the left temporal and frontal areas and in the right central area (F7, T3, F7-T3, C4). The ictal EEG recorded an asymmetric tonic seizure with a right-sided emphasis and clonic movements in the right leg. The EEG showed regional slowing in the left fronto-central region, with the appearance of repetitive fast activity in the left frontal area (F3). Subsequent EEGs demonstrated a progressive increase in epileptiform activity.

**FIGURE 1:**
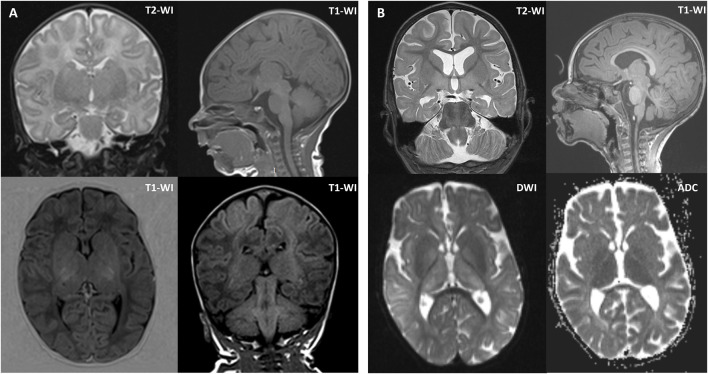
Brain MRI of the proband at 1 month of age **(A)** and 2 years and 1 month of age **(B)**. **(A)** Brain MRI at 1 month of age showed symmetrically increased MR signal and volume of the putamen and thalamus. **(B)** Brain MRI at 2 years and 1 month of age showed mild diffuse cerebral atrophy without cortical architectural disruption, except for hypoplasia of the corpus callosum, periventricular white matter alterations, hypomyelination of the deep white matter, and symmetrical enlargement of the putamen, thalamus, and red nucleus. Axial diffusion-weighted imaging (DWI) and apparent diffusion coefficient (ADC) images showed restricted diffusion in the basal ganglia. Coronal T2-weighted images (T2-WI) at 1 month of age and 2 years and 1 month of age revealed mild progression of cerebral atrophy. Sagittal T1-weighted images (T1-WI) at 1 month of age and 2 years and 1 month of age showed mild progression of hypoplasia of the corpus callosum.

Brain magnetic resonance imaging (MRI) was performed twice. The first MRI, performed at 1 month of age, showed increased MR signals and volume in the putamen and thalamus bilaterally. The second MRI, performed at 2 years and 1 month of age, revealed atrophy of the cerebral cortex and corpus callosum, a persistent lack of myelination, and periventricular leukopathy, hypomyelination of the deep white matter, and symmetrical enlargement of the putamen, thalamus, and red nucleus (Figure 1). A comparison of the two scans showed mild progression of cerebral atrophy and hypoplasia of the corpus callosum.

A positive clinical response was observed with ethosuximide therapy, which provided 9 months of remission. Vigabatrin led to 1 month of remission, followed by a reduction in seizure frequency.

### 3.2 Genetic and functional studies

Gene panel sequencing identified two heterozygous missense variants in the *ADSL* gene, confirmed by segregation analysis to be in trans position. The first variant, c.340T>C (p.Tyr114His) [Clinvar ID:204807], is a previously described variant with functional studies indicating no residual enzyme activity in the mutant protein (Kmoch et al., 2000; Zikanova et al., 2010). The second, a novel missense variant c.859A>G (p.Ile287Val) [Clinvar ID:1437877], is found in the gnomAD v.4.1 database with a low frequency (0.000004337, 7 heterozygous alleles) (Chen et al., 2024). The majority of *in silico* programs classify this missense variant as benign or of uncertain significance. However, according to spliceAI predictions, the c.859A>G variant could create a new donor splice site (Donor Gain 0.98) (Jaganathan et al., 2019).

**FIGURE 2:**
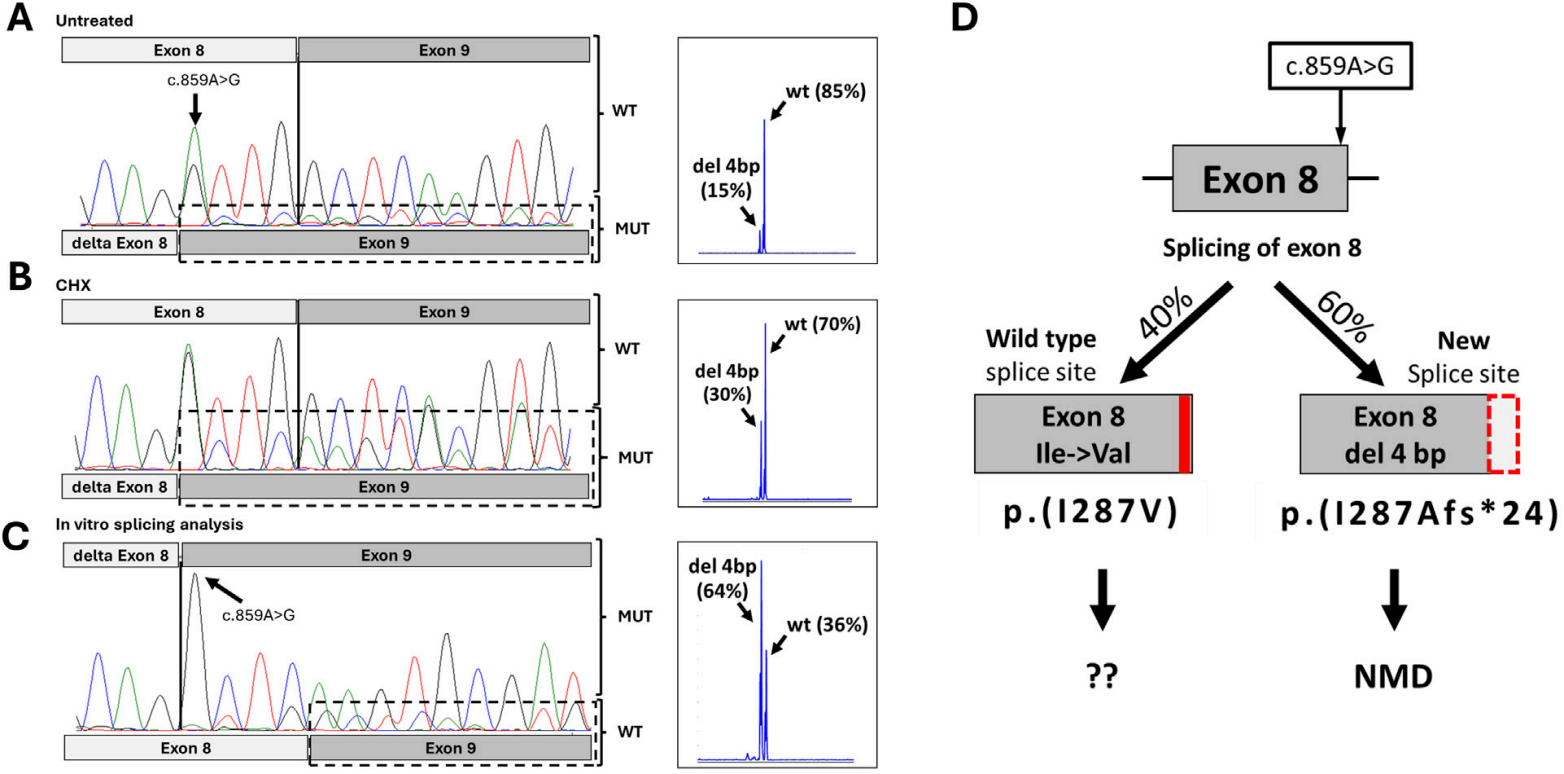
Results of functional studies for c.859A>G. **(A)** Sanger chromatogram and fragment analysis results from the proband’s peripheral blood mononuclear cells. **(B)** Sanger chromatogram and fragment analysis results from the proband’s peripheral blood mononuclear cells after inhibition of NMD. **(C)** Sanger chromatogram and fragment analysis results from minigen construction with variant c.859A>G. **(D)** Schematic representation of the possible splicing effects caused by the c.859A>G variant, based on splicing analysis results.

To investigate the possible impact of the variant on splicing, we performed RT-PCR analysis of the patient’s mRNA, extracted from blood. Sequencing of the RT-PCR product of the proband revealed two isoforms: a major one corresponding to the wild type, and a minor one with exon 8 shortened by 4 bp. Fragment analysis showed that the expression of the wild-type *ADSL* mRNA isoform was 85%, while the expression of the aberrant isoform was 15% (Figure 2A). The same allelic imbalance was observed in the mRNA sample from the patient’s mother. Because the proband had a different single-nucleotide variant (SNV) in the trans position, we sequenced the locus containing the c.340T>C variant in both the proband and the proband’s carrier father. Sequencing showed that the father is fully heterozygous for this variant, while the proband exhibited a reduced level of the wild-type allele by approximately 50%.

Given that the mutant isoform with the 4-nucleotide deletion is expected to result in a frameshift and a premature stop codon, we hypothesized that the mutant isoform carrying the c.859A>G variant undergoes nonsense-mediated mRNA decay (NMD). To test this, we inhibited NMD by treating the patient’s PBMCs with cycloheximide for 6 h. Sequencing of RT-PCR products following NMD inhibition revealed an increase in the mutant isoform peaks to approximately 30%, achieving complete heterozygosity at position c.859 (Figure 2B). Subsequent fragment analysis confirmed that expression of the mutant isoform rose to 30% (Figure 2B). This observed signal change after NMD inhibition suggests that the newly formed donor splicing site is not utilized in 100% of cases, allowing some transcripts to be synthesized using the wild-type donor site.

To verify this, we conducted an *in vitro* splicing assay. HEK293T cells were transfected with plasmids containing the *ADSL* gene region surrounding exons 8 and 9, with or without the c.859A>G variant. After 48 h, total RNA was isolated, cDNA was synthesized, and RT-PCR with sequencing was performed. The results showed that the c.859A>G variant leads to the creation of a new donor splice site and a 4-bp deletion in exon 8, as predicted. However, the wild-type isoform was also present. Fragment analysis indicated that in a monoallelic expression system, the wild-type and mutant isoforms comprised approximately 36% and 64%, respectively (Figure 2C).

In summary, the c.859A>G variant leads to the production of two types of transcripts. The primary event (∼60%) is the formation of a new splicing donor site with a 4-bp deletion in exon 8, causing a frameshift and premature stop codon (p.Ile287Alafs*24). This transcript undergoes NMD and does not contribute to protein synthesis (Figure 2D). However, in approximately 40% of cases, the spliceosome utilizes the wild-type donor site, resulting in full-length mRNA with the missense variant Ile287Val.

## 4 Discussion

Here, we reported a clinical case of ALD type I with two pathogenic variants (p.Ile287Val and p.Tyr114His) in the *ADSL* gene. The variant c.859A>G (p.Ile287Val) was predicted to create a new donor splice site. Because the second variant, c.340T>C (p.Tyr114His), is located in the enzyme’s active site and has no residual enzyme activity, and biallelic loss-of-function variants are typically lethal, we suggested that the c.859A>G (p.Ile287Val) variant retains some residual enzyme function. Our study demonstrates that this variant produces two types of mutant transcripts. The primary effect is the activation of a new donor splice site in 64% of transcripts, which undergo NMD. The remaining 40% of transcripts produce a mutant ADSL protein isoform with the Ile287Val substitution.

In previous studies, the p.Tyr114His variant had been identified in 14 patients with clinical data (Kmoch et al., 2000; Mouchegh et al., 2007; Jurecka et al., 2008; Mastrangelo et al., 2019; Mastrogiorgio et al., 2021; Cutillo et al., 2024). Of these patients, seven were diagnosed with the neonatal form of ALD, with age of death under 4 months; six were diagnosed with ALD type I; and only two patients with compound-heterozygous Gly418Ala had type II ALD (Table 1). In two studies, residual ADSL activity in fibroblasts was measured, showing 2%–23% S-AMP and 6%–20% SAICAr metabolism for patients with the neonatal form and 37%–39% S-AMP and 46% SAICAr metabolism for those with ALD type I (Kmoch et al., 2000; Mouchegh et al., 2007).

**TABLE 1:** Published patents with Y114H in a compound-heterozygous state with other variants.

Source	SNV in a compound-heterozygous state with Y114H/Clinvar ID:204807	ALD spectrum	Sex	Onset	S-ADO/SAICAr ratio		Residual ADSL activities in fibroblasts	
					Urine	CSF		
							S-AMP, %	SAICAR, %
Mouchegh et al. (2007)	R426H/Clinvar ID:2462	Neonatal form with AoD - 12 weeks	M	Neonatal	ND	ND	ND	ND
Mouchegh et al. (2007)	R426H/Clinvar ID:2462	Neonatal form with AoD - 6 weeks	M	Neonatal	ND	ND	ND	ND
Mouchegh et al. (2007)	R396C/Clinvar ID:838200	Neonatal form with AoD - 6 weeks	M	Neonatal	ND	ND	16	ND
Mouchegh et al. (2007)	R426H/Clinvar ID:2462	Neonatal form with AoD - 6 weeks	F	Neonatal	ND	0,5	23	20
Mouchegh et al. (2007)	R426H/Clinvar ID:2462	Neonatal form with AoD - 8 days	M	Neonatal	1,1	ND	2	6
Mouchegh et al. (2007)	E376D/Clinvar ID:204795	Neonatal form with AoD - 9 weeks	M	Neonatal	0,7	0,6	8	10
Jurecka et al. (2008)	T242I/Clinvar ID:803695	Neonatal form with AoD - 9 weeks	F	Neonatal	ND	ND	ND	ND
Jurecka et al. (2008)	T242I/Clinvar ID:803695	Type I	F	Third week	1,1	0,9	ND	ND
Mastrangelo et al. (2019)	A22V/ND	Type I	M	Neonatal	6,3	ND	ND	ND
Kmoch et al. (2000)	R190Q/Clinvar ID:2465	Type I	M	4 months	1,2	ND	39	46
Kmoch et al. (2000)	R190Q/Clinvar ID:2465	Type I	F	6 months	1,1	ND	37	46
Current study	I287V/Clinvar ID:1437877	Type I	M	1 month	ND	ND	ND	ND
Mastrogiorgio et al. (2021)	G418A/ND	Type I	F	6 months	ND	ND	ND	ND
Mastrogiorgio et al. (2021)	G418A/ND	Type II	M	6 month	3,6	ND	ND	ND
Mastrogiorgio et al. (2021)	G418A/ND	Type II	M	6 months	3,7	ND	ND	ND
Cutillo et al. (2024)	R296W/Clinvar ID:450065	Type II^a^	M	18 months	ND*	ND	ND	ND

^a^ based on clinical data from the article. AoD, Age of death; CSF, Cerebrospinal fluid; SNV, Single-nucleotide variant.

**FIGURE 3:**
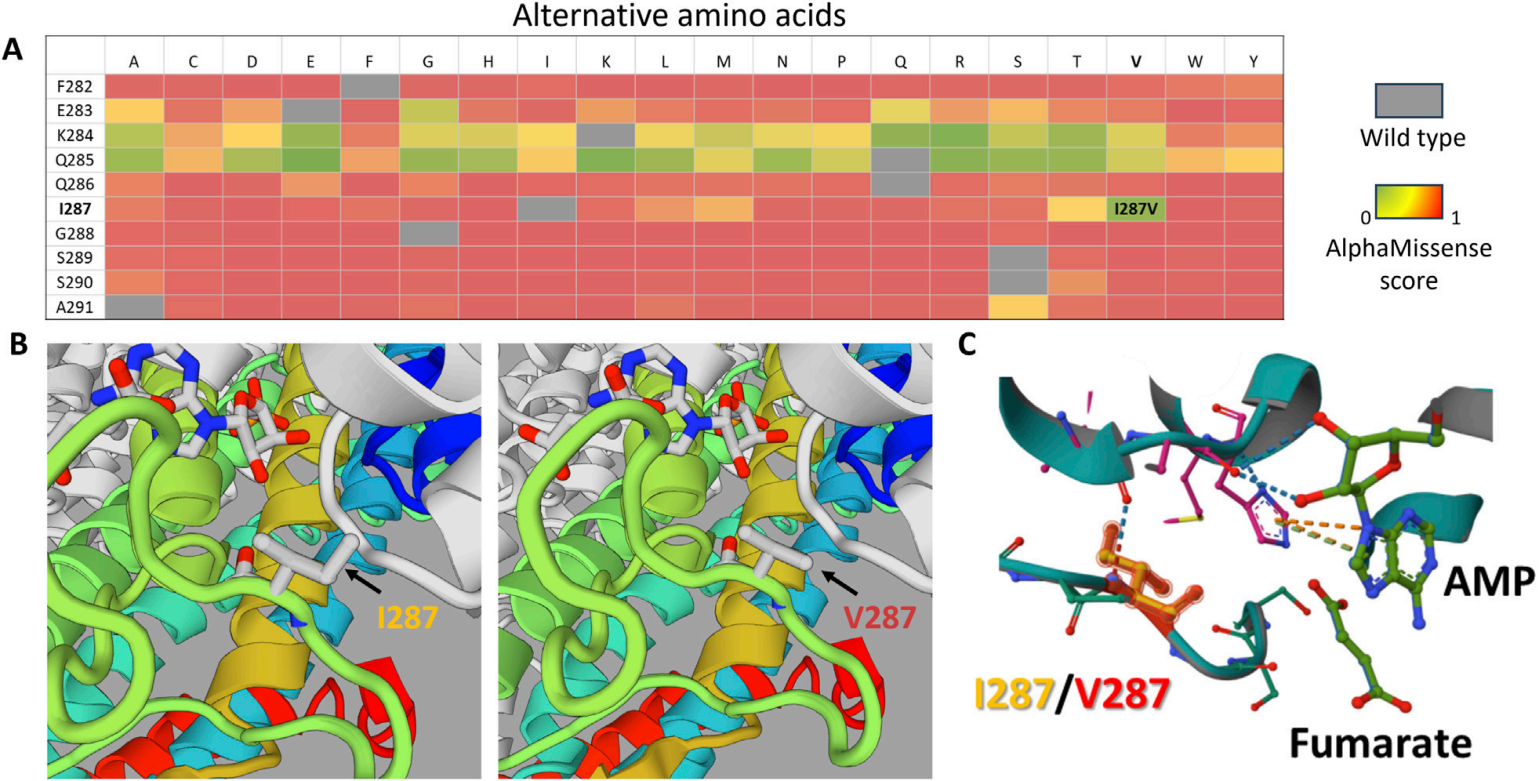
*In silico* analysis of the p.Ile287Val substitution. **(A)** Heatmap based on AlphaMissense scores for all possible amino acid substitutions in the F282–A291 region of the ADSL protein. Scores close to 0 (green) indicate substitutions with minimal predicted impact on protein structure, whereas higher scores indicate potential structural effects. **(B)** 3D model comparison of the wild-type protein (left) and the mutant protein (right), highlighting the region surrounding position 287. **(C)** Structural alignment of the mutant (turquoise) and wild-type (gray) protein models, demonstrating that position 287 is close to the enzyme’s active site, which is potentially important for enzyme function.

Our proband presented with symptoms consistent with type I ALD and had approximately 40% of transcripts containing the Ile287Val variant from the mutant allele, while the other allele carried the p.Tyr114His variant, which is known to have zero residual enzyme activity (Kmoch et al., 2000; Zikanova et al., 2010). Previous studies have shown that some residual ADSL activity is essential for non-lethal phenotypes (Dutto et al., 2022). According to AlphaMissense predictions, substitutions at codon 287 could most likely disrupt the ADSL wild-type structure (Figure 3A). However, the substitution to valine (Ile287Val) had the lowest pathogenicity score (0.16) in this position, according to AlphaMissense (Figure 3A), and other *in silico* predictions classified it as likely benign (Jumper et al., 2021).

Through 3D modeling of the Ile287Val isoform, we found no significant structural differences compared to the wild type (Figures 3B,C). However, based on *in silico* predictions alone, we cannot rule out the possibility that Ile287Val may have reduced activity compared to the wild-type isoform. If the Ile287Val variant functions similarly to the wild type in terms of enzymatic activity, then the question arises as to whether it could cause any diseases within the ALD phenotype spectrum in a homozygous state.

One limitation of our study is the lack of enzyme activity testing, which could provide more insight into pathogenicity and its impact on succinylaminoimidazolecarboxamide ribose-5′-phosphate (SAICAR) or S‐AMP enzymatic activity. Testing could also clarify whether certain variants contribute to increased SAICAR or S-AMP production compared to the wild type. Additionally, we examined other genes known to be involved in *de novo* purine synthesis using an MAF <0.5% filter. Although we did not measure the S-Ado/SAICAR ratio, we believe that the clinical presentation, combined with our genetic findings, is sufficient to diagnose the proband with ADSL deficiency.

## 5 Conclusion

Our case involved a type I ALD patient with two pathogenic variants in the *ADSL* gene: p.Ile287Val and p.Tyr114His. The c.859A>G (p.Ile287Val) variant leads to abnormal splicing, resulting in transcripts that undergo NMD, while others produce a potentially functional protein with the p.Ile287Val variant. Although enzyme activity testing was not performed, the clinical presentation and genetic findings strongly support the diagnosis of ALD type I.

## Data Availability

The data generated and analyzed in this study are not publicly available due to ethical restrictions and institutional policies regarding patient confidentiality. The informed consent obtained from participants does not allow for public data sharing, in compliance with the approved protocol by the ethics committee. However, the data may be made available to qualified researchers upon reasonable request and with appropriate ethical approvals.
