# Supplementary material for: Case Report: Adenylosuccinate lyase deficiency type I caused by splicing disruption due to a novel missense variant in the ADSL gene
Source: Front Genet. 2025 Oct 24;16:1670299. doi: 10.3389/fgene.2025.1670299 (PMC12591842; doi:10.3389/fgene.2025.1670299)
Supplement: Supplementary file 1 [file DataSheet1.docx]

**Brief summary of CARE checklist**

The manuscript’s title clearly states the primary diagnosis and includes “case report.” Keywords incorporate disease names and the phrase “case report.” The abstract highlights the uniqueness of the case—a novel missense variant causing splicing alteration—alongside the patient’s clinical presentation, diagnosis via whole-exome sequencing and RNA analysis, treatment attempts, and conclusions emphasizing RNA-level analysis. The introduction summarizes the novelty and clinical significance of the findings. Patient information, clinical findings, and diagnostic assessment are detailed in the manuscript, including seizure onset, neurological symptoms, EEG and MRI results, and genetic findings. Therapeutic interventions and outcomes are described, noting temporary seizure control but ongoing neurodegeneration. Discussion covers strengths and limitations, relevant literature, rationale for conclusions, and the main takeaway on the importance of transcript-level analysis. Patient perspective is unavailable due to age and condition, and informed consent was obtained from the patient’s legal representative.

**The CARE Checklist**

1. **Title – The diagnosis or intervention of primary focus followed by the words “case report”.**The title includes both the primary diagnosis and the phrase “case report.”
2. **Key Words**
   We included "case report" along with disease names and methods in the keywords: “Keywords: ADSL, ALD, adenylosuccinate lyase deficiency, case report, splicing”
3. **Abstract – (structured or unstructured)**
   1. **Introduction – What is unique about this case and what does it add to the scientific literature?**

We describe the first ALD case caused by a missense variant leading to splicing alteration.

- 1. **The patient’s main concerns and important clinical findings.**

The patient, a 2-year-old boy, presented with early-onset polymorphic seizures (clonic and myoclonic), developmental delay, and progressive neurological deterioration. Seizures were initially resistant to treatment but showed temporary control with ethosuximide and vigabatrin. Neurodegeneration continued despite therapy.

- 1. **The primary diagnoses, interventions, and outcomes**

The primary diagnosis was adenylosuccinate lyase deficiency (ALD), confirmed by analysis of whole-exome sequencing and RNA studies revealing compound heterozygous variants in the ADSL gene. Interventions included antiseizure therapy with ethosuximide and vigabatrin, which achieved temporary seizure control. Despite treatment, the patient experienced continued neurological decline, consistent with severe type I ALD

- 1. **Conclusion – What are one or more “take-away” lessons from this case report?**

Our case illustrates that RNA-level analysis can reveal pathogenic splicing effects of variants predicted to be benign at the protein level, emphasizing the value of transcriptome studies in rare disease diagnostics.

1. **Introduction**
   1. **Briefly summarizes why this case is unique and may include medical literature references.**

This report describes the first ALD-associated variant c.859A>G (p.Ile287Val) that acts through splicing disruption and NMD, contrasting with previously reported protein-affecting variants. We establish RNA-level effects as a novel disease mechanism in ALD.

1. **Patient Information**
   1. **De-identified patient specific information.**

Presented in section 3.2.1 Clinical presentation - see main manuscript:

“The proband was a 2-year-old boy (first known affected family member) with a history of clonic, myoclonic, and generalized seizures. He was born at term following his mother's first pregnancy, with a birth weight of 3500 g, body length of 52 cm, and Apgar scores of 8 and 9 at 1 and 5 minutes, respectively. By the end of the first month, he exhibited poor sucking reflexes and minimal weight gain (only 90 g).”

- 1. **Primary concerns and symptoms of the patient.**

Presented in section 3.2.1 Clinical presentation - see main manuscript:

“Seizures began at one month of age, with polymorphic manifestations, including clonic, myoclonic, and generalized seizures, occurring up to five times daily.”

- 1. **Medical, family, and psychosocial history including relevant genetic information.**

Presented in section 3.2.1 Clinical presentation - see main manuscript:

“The proband was a 2-year-old boy (first known affected family member) with a history of clonic, myoclonic, and generalized seizures.”

- 1. **Relevant past interventions and their outcomes.**

1. **Clinical Findings**
   1. **Describe significant physical examination (PE) and important clinical findings.**

Presented in section 3.2.1 Clinical presentation - see main manuscript:

At 2 years of age, the proband's developmental indicators remained below normal (height: 79 cm [-2.35 SD], weight: 9.5 kg [-2.60 SD], head circumference: 45 cm [-2.56 SD]). Clinical examination showed muscular dystonia, hyperreflexia in both upper and lower limbs, and hypersalivation.

1. **Timeline – Historical and current information from this episode of care organized as a timeline (figure or table).**

NA

1. **Diagnostic Assessment**
   1. **Diagnostic methods (PE, laboratory testing, imaging, surveys).**

Presented in section 3.2.1 Clinical presentation - see main manuscript:

“Video electroencephalography (EEG) at 1 month of age revealed multiregional epileptiform activity and diffuse discharges. During the recording, an asymmetric tonic seizure was observed, predominantly involving the right-sided limbs, followed by a clonic seizure in the right leg. Subsequent EEGs demonstrated a progressive increase in epileptiform activity. Brain magnetic resonance imaging (MRI) was performed twice: at 1 month, MRI showed delayed myelination; by 2 years of age, MRI revealed cerebral cortex and corpus callosum atrophy, persistent lack of myelination, and periventricular leukopathy.”

- 1. **Diagnostic challenges.**
  2. **NA**
  3. **Diagnosis (including other diagnoses considered).**
  4. **NA**
  5. **Prognostic characteristics when applicable.**

NA

1. **Therapeutic Intervention**
   1. **Types of therapeutic intervention (pharmacologic, surgical, preventive).**

Pharmacologic. Presented in section 3.2.1 Clinical presentation - see main manuscript:

A positive clinical response was observed with Ethosuximide therapy, which provided 9 months of remission. Vigabatrin led to 1 month of remission, followed by a reduction in seizure frequency.

- 1. **Administration of therapeutic intervention (dosage, strength, duration).**

**NA**

- 1. **Changes in therapeutic interventions with explanations.**

**NA**

1. **Follow-up and Outcomes**
   1. **Clinician- and patient-assessed outcomes if available.**

Seizures were initially resistant to treatment but showed temporary control with ethosuximide and vigabatrin. Neurodegeneration continued despite therapy.

- 1. **Important follow-up diagnostic and other test results.**

NA

- 1. **Intervention adherence and tolerability. (How was this assessed?)**

NA

- 1. **Adverse and unanticipated events.**

NA

1. **Discussion**
   1. **Strengths and limitations in your approach to this case.**

A major strength of this case is the combined use of genomic (whole-exome sequencing) and transcriptomic (RNA) analysis, which enabled identification of a cryptic splicing defect that would have been missed by in-silico protein-level prediction alone. This integrative approach revealed the true pathogenicity of the novel c.859A>G variant. A limitation of our study is the absence of functional enzyme activity testing, which could have clarified the precise biochemical impact of the p.Ile287Val isoform on ADSL function and metabolite levels.

- 1. **Discussion of the relevant medical literature.**

Previous reports describe the p.Tyr114His variant as associated with severe, often neonatal, forms of ALD, with no residual enzyme activity. Our findings align with earlier literature suggesting that even minimal residual ADSL activity can prevent a lethal phenotype. Clinical and metabolic data from similar patients in the literature support our interpretation that the p.Ile287Val variant provides partial function, consistent with a type I ALD phenotype. In-silico analyses and structural modelling support the hypothesis that Ile287Val may preserve some enzymatic activity, though definitive conclusions require functional validation.

- 1. **The rationale for your conclusions.**

Our conclusion is based on the alignment of clinical presentation with molecular findings, including compound heterozygosity involving a known null allele (p.Tyr114His) and a novel variant (p.Ile287Val) that induces aberrant splicing. RNA analysis revealed that approximately 36–40% of transcripts from the mutant allele produce an mRNA with the p.Ile287Val substitution. Given the severe phenotype but absence of a lethal neonatal presentation, we propose that the p.Ile287Val isoform retains near-normal function, and that this residual expression is sufficient to support survival beyond infancy.

- 1. **The primary “take-away” lessons from this case report (without references) in a one paragraph conclusion.**

This case highlights the importance of transcript-level analysis when interpreting variants of uncertain significance, particularly those predicted to be benign on the protein level. It also demonstrates how splicing effects can underlie pathogenicity in cases where no clearly damaging protein-coding change is apparent. Integrating clinical, genomic, and RNA data can reveal splicing alterating mechanisms that support diagnosis in rare metabolic disorders when functional enzyme testing is unavailable.

1. **Patient Perspective – The patient should share their perspective on the treatment(s) they received.**

Not available due to the patient’s young age and neurological condition, which precluded reliable communication of personal perspectives.

1. **Informed Consent – The patient should give informed consent. (If not, explain)**

The patient’s legal representative provided written informed consent specifically for publication of this case report.
